# Acute benign pleural effusion, a rare presentation of hepatitis A virus: a case report and review of the literature

**DOI:** 10.1186/s13256-022-03449-w

**Published:** 2022-06-09

**Authors:** Jihad Samer Zalloum, Tareq Z. Alzughayyar, Fawzy M. Abunejma, Ibba Mayadma, Layan Ziad Tomeh, Karim Jamal Abulaila, Asil Husam Yagmour, Khalid Jamal Faris, Mohammed A. S. Aramin, Mo’min Ra’id Mesk, Asala Khalil Hasani, Balqis Mustafa Shawer, Rawand Hisham Titi, Ayat A. Z. Aljuba, Hussam I. A. Alzeerelhouseini, Yousef I. M. Zatari

**Affiliations:** 1 Faculty of Medicine, Al-Quds University, Main Campus, P.O. Box 89, Abu Dis, Palestine; 2 Al-Ahli Hospital, Hebron, Palestine; 3 Faculty of Medicine, Palestine Polytechnic University, Hebron, State of Palestine; 4 Faculty of Medicine & Health Sciences, An-Najah National University, Nablus, Palestine

**Keywords:** Pleural effusion, Ascites, Acute hepatitis, Acute hepatitis A virus, HAV associated with self-limited pleural effusion, Unusual manifestation, Conservative management

## Abstract

**Introduction:**

Hepatitis A virus infections are mostly asymptomatic or mildly symptomatic, and generally this disease has a benign course and resolves spontaneously. However, intrahepatic and rarer extrahepatic manifestations can complicate typical cases of acute hepatitis. Pleural effusion is an extremely rare extrahepatic entity with 20 cases reported in literature.

**Case presentation:**

We report herein a recent case of both pleural effusion and ascites accompanying hepatitis A infection in a 5-year-old middle eastern child, diagnosed using serological testing and imaging studies, who was treated with supportive management with full resolution after 2 weeks. In addition, we review available literature regarding hepatitis A virus associated with pleural effusion using PubMed and summarize all reported cases in a comprehensive table.

**Results:**

Literature contains 20 reported cases of serology-confirmed hepatitis A virus presenting with pleural effusion, most in the pediatric population with average age at presentation of 9 years 8 months. The majority of reported patients had right-sided pleural effusion (50%) or bilateral effusion (45%), while only 5% presented with pleural effusion on the left side. Hepatomegaly and ascites occurred concurrently in 80% and 70% respectively. Supportive treatment without invasive procedures (except one chylothorax case) yielded complete recovery in 95% of cases, while only one case progressed to fulminant liver failure followed by death.

**Conclusion:**

Acute hepatitis A virus rarely presents with pleural effusion, usually following a benign course with spontaneous resolution in most patients. Pleural effusion does not change the prognosis or require any invasive treatment. Thus, further invasive procedures are not recommended and would only complicate this self-resolving benign condition.

## Introduction

Hepatitis A virus (HAV) is a positive-stranded Ribonucleic acid (RNA) virus that is stable at moderate temperatures and low pH, allowing for prolonged survival in the environment and fecal–oral transmission. It is known to circulate among children, especially in developing countries due to poor hygiene and lack of sanitation [1]. Although hepatitis A is usually asymptomatic or presents with mild symptoms in children, extrahepatic manifestations and, particularly, pleural effusions are rare [2, 3]. The first case of pleural effusion caused by hepatitis A as underlying infection was described as early as 1971 by Gross and Gerding [4], but this association has been scarcely reported in medical literature, with no more than 20 cases [5]. We provide herein a comprehensive literature review of 20 published cases and also report a new case, to clarify this rare entity.

## Case presentation

A previously healthy 5-year-old middle eastern boy with no known history of any medical diseases presented to the emergency department with jaundice and scleral icterus, in addition to dark-colored urine, abdominal pain and distention, and slight shortness of breath beginning 4 days previously after contact history with individuals having acute hepatitis A symptoms.

He had no previous history of traveling, blood transfusion, bleeding, or previous medical, drug, or surgical treatment.

Upon presentation, during physical examination, the patient had high fever (39 °C), abdominal distention, hepatomegaly with normal spleen size, unilateral basal right-sided decreased breathing sound and dullness, as well as tachycardia and tachypnea. The rest of the examination was normal, including normal mental status.

**Fig. 1:**
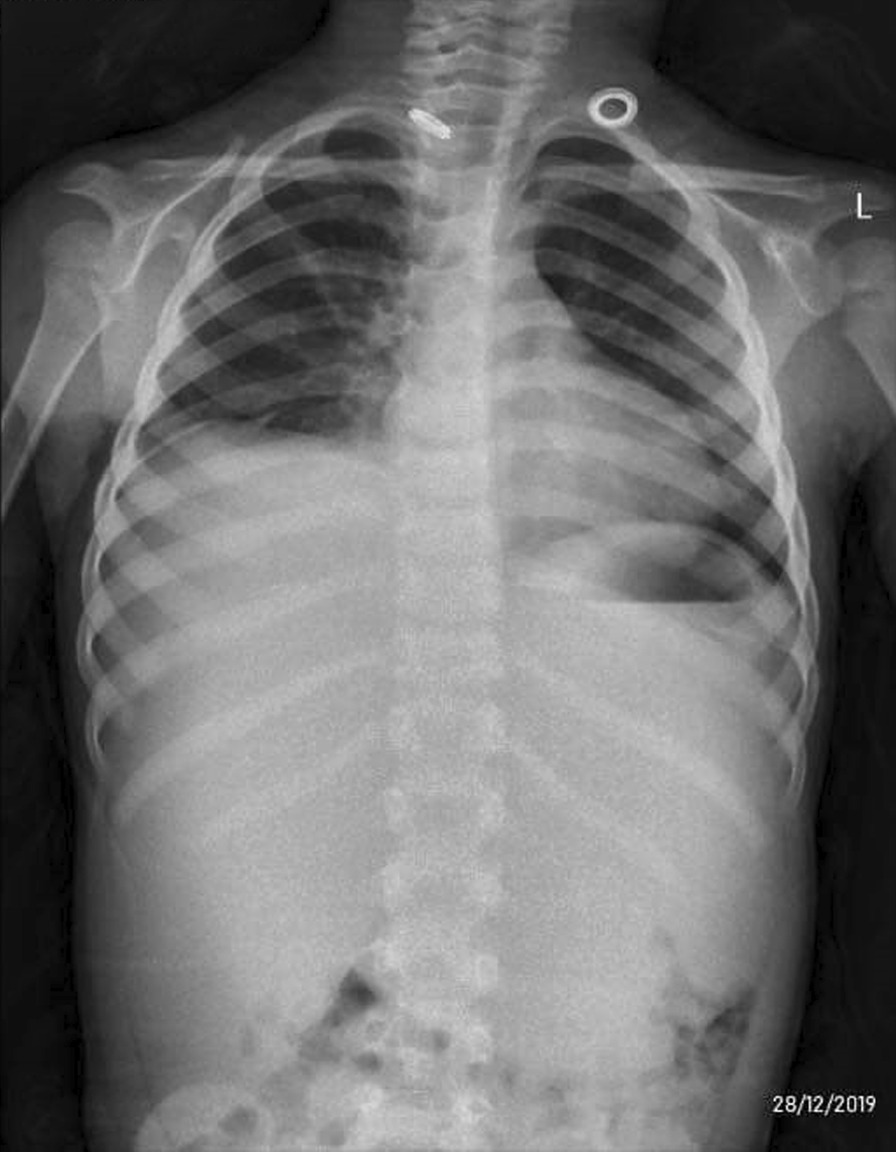
Chest x-ray showing right-sided pleural effusion

**Fig. 2:**
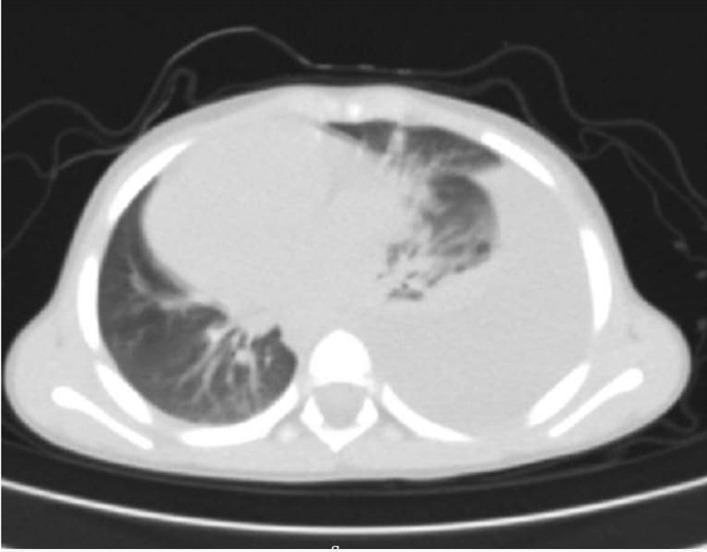
Computed tomography showing right-sided pleural effusion

The patient was admitted, and laboratory investigations were carried out (Tables 1, 2). HAV serology testing was positive. Chest x-ray showed unilateral right-sided pleural effusion. Chest contrast computed tomography (CT) scan delineated right effusion with significant lung collapse, plus negligible amount on the left side and clear left lung field (Figs. 1, 2). Abdominal sonography and abdominal–pelvic CT scan identified hepatomegaly and ascites. Echocardiography was free of any abnormality.

**Table 1:** Laboratory analysis

Laboratory analysis	Result
Hb	11.7 g/dl
WBC	6.5 × 10^3^ cells/mm^3^
PLT	230 × 10^3^/mm^3^
Serology HAV Igm	+ve HAV IgM −ve indirect Coombs
PT	14.4 (12.3 control)
PTT	25 (26 control)
INR	1.17
Albumin	2.9 g/dl
GGT	101 U/l
ALP	410 IU/l
Serum ammonia	115 UG/dl
Coombs test	Negative

Hb: Hemoglobin, WBC: White blood cells, PLT: platelets, PT: Prothrombin Time, PTT: Partial Thromboplastin Time, INR: International normalized ratio, GGT: amma-glutamyl transferase, ALP: alkaline phosphatase, BUN: Blood urea nitrogen

Urinalysis also normal

**Table 2:** Liver biochemical markers tracing during hospital stay

Liver biochemical markers tracing during hospital stay	1st day	2nd day	3rd day
AST (U/l)	470	396	300
ALT (U/l)	883	654	578
Total bilirubin (mg/dl)	8.9	4.1	4.1
Direct bilirubin (mg/dl)	6.6	2.7	3

AST: Aspartate aminotransferase, ALT: alanine aminotransferase

On day 5 post-discharge, follow-up LFTs normalized

Our patient was diagnosed with HAV acute hepatitis associated with right-side pleural effusion and ascites, confirmed by CT scan. Treatment consisted of supportive parenteral fluid and carbohydrate-enriched diet, while no diuretics or antibiotics were used. The patient was discharged on day 4 after significant improvement, achieving full clinical and biochemical recovery 5 days postdischarge with normal liver function tests and normal lung and abdominal imaging.

## Discussion

Acute hepatitis caused by hepatitis A virus infection can manifest with a variety of symptoms and severities. One important factor is age, as disease severity is inversely proportional to age, with more than 80% of children having a less severe course and complete recovery within 3 months, usually being asymptomatic and anicteric. However, severity and mortality rates increase with advancing age [6].

Onset of symptoms follows a mean incubation period of approximately 30 days. Common signs and symptoms include fever, jaundice, fatigue, abdominal pain, nausea, and emesis.

Infectivity and viral shedding last from the beginning of the incubation period until 1 week after jaundice resolution, during which the virus is capable of fecal–oral spread [7].

HAV acute hepatitis may be associated with many complications, including:

1. Intrahepatic: such as cholestatic hepatitis, relapsing hepatitis, and autoimmune hepatitis. Rarely, hepatitis A can progress to acute liver failure.
2. Extrahepatic manifestations are infrequently reported in HAV acute hepatitis (6.4–8%) and may include: urticarial and maculopapular rash, acute kidney injury, autoimmune hemolytic anemia, aplastic anemia, acute pancreatitis, mononeuritis, reactive arthritis, Guillain–Barre syndrome and pleural or pericardial effusion, ascites, glomerulonephritis, polyarteritis nodosa, cryoglobulinemia, and thrombocytopenia [2, 3, 8].

Among those complications, pleural effusion is an extremely rare co-occurring condition that is scarcely reported in literature. The exact mechanism is unknown and could be multifactorial. There are many theories regarding the suspected pathogenesis of this entity:

1. Kurt *et al.* suggested direct viral invasion of pleura, immune complex deposition, or inflammatory response as probable cause, since HAV ribonucleic acid was found in the pleural fluid of a HAV viral hepatitis patient by polymerase chain reaction [9].
2. Dhakal *et al.* postulated that copresenting ascites could contribute to the pleural effusion via small diaphragmatic defects or diaphragmatic lymphatics [5, 10].
3. Also, a decrease in the plasma oncotic pressure as well as a transient rise of the pressure in the portal vein and/or lymphatics due to compression by the hepatic sinusoids may be a contributory factor in some cases developing ascites and pleural effusion [5, 11].

Tables 3 and 4 present a comprehensive review of available published cases of HAV with pleural effusion. All 20 patients were diagnosed by positive serum anti-HAV IgM antibodies and pleural effusion on imaging studies, regardless of the underlying nature of effusion (transudative or exudative). Most patients were from the pediatric population with average age of 9 years 8 months, while 80% (16 patients) were younger than 12 years old, with a male-to-female ratio of 9:11. Most patients presented with usual symptoms of acute hepatitis including fever, vomiting, abdominal pain, jaundice, icterus, and fatigue. Also, abdominal and chest examination revealed hepatomegaly, abdominal distention, chest dullness, and decreased airway entry and normal mental status in all patients. Laboratory testing showed an average of 3.1 albumin g/dl, with average total and direct bilirubin of 5.2 and 4 mg/dl respectively. Chest x-ray, ultrasonography, and in some patients computerized tomography or magnetic resonance imaging all generally showed similar results: most patients had right-sided (ten patients) or bilateral pleural effusion (nine patients), while one case had effusion on the left side. Also, the majority copresented with hepatomegaly (16 patients) and ascites (14 patients). Finally, thickened gallbladder wall was seen in only three patients. Moreover, pleural fluid analysis mostly showed a transudative nature of the effusion, while one patient had exudative effusion resulting from *Salmonella paratyphi* A superinfection, and one case had chylothorax, but pleural fluid analysis was carried out in a limited number of patients (nine).

**Table 3:** Literature review

Study	Sex	Age	Chief compliant	Physical examination	Management	Diuretics	Outcome
Saha [15]	M	3 years	Generalized body swelling	Icterus, bilateral pitting edema, abdominal distention, hepatomegaly	Supportive management	N/A	Spontaneous resolution after 4 days
Roy [16] Case 1	F	6 years	Fever, vomiting, fatigue	Icterus, abdominal distention, hepatomegaly, decreased breath sounds on the right side of chest	Supportive management, B-complex, ursodeoxycholic acid, oral lactulose	++	Spontaneous resolution after 1 week
Roy [16] Case 2	M	4 years	Fever, jaundice	Abdominal distention, hepatomegaly	Supportive management	++	Spontaneous resolution
Owen [17]	M	42 years	Fever, malaise, pleuritic pain	Dullness on the base of right lung	Supportive management	N/A	Spontaneous resolution
Dalai *et al.* [14]	F	3 years	Fever, abdominal pain	Icterus, hepatomegaly	Supportive management, IV vit. K, oral antibiotic	N/A	Spontaneous resolution after 3 weeks
Nagarajan *et al.* [18] Case 1	F	7 years	Abdominal pain, jaundice	Icterus, hepatomegaly, decreased breath sounds on the right side of chest	Supportive management	N/A	Spontaneous resolution after 3 weeks
Nagarajan *et al.* [18] Case 2	F	10 years	Fever, vomiting, abdominal pain	Icterus, hepatomegaly	Supportive management	N/A	Spontaneous resolution
Allen *et al.* [19]	F	30 years	Flu like symptoms, fatigue, myalgia	Icterus, right upper quadrant abdominal tenderness	Supportive management	N/A	Spontaneous resolution
Selimoğlu *et al.* [20]	M	8 years	Fever, jaundice, anorexia, abdominal pain	Icterus, hepatomegaly, dullness on the base of right lung	Supportive management	N/A	Spontaneous resolution
Mehta *et al.* [13]	M	3 years	Fever, vomiting, abdominal pain, jaundice	Icterus, hepatomegaly, dullness on the base of right lung	Supportive management, IV amoxicillin–clavulanic acid, thoracotomy with chest tube insertion	N/A	Complete resolution after 1 week
Alhan *et al.* [14]	M	3 years	Fever, vomiting, jaundice	Febrile, icterus, hepatomegaly, dullness on the base of right lung	Supportive management	N/A	Death after 2 weeks due to fulminant liver failure, increased intracranial pressure
Erdem *et al.* [10]	M	12 years	Nausea, vomiting, fatigue	Icterus, febrile, hepatomegaly	Supportive management, vit. K, protein/lipid–restricted and carbohydrate-enriched diet	N/A	Spontaneous resolution after 10 days
Ghosh and Kundu [12]	F	4 years	Fever, jaundice, cough, dyspnea	Hepatomegaly, splenomegaly, abdominal distention, dullness on the base of right lung	Supportive management, IV vit. K, IV cefotaxime	N/A	Complete resolution after 1 week
Gürkan *et al.* [10]	M	4 years	Jaundice, abdominal pain, vomiting, headache	Jaundice, febrile, abdominal distention, hepatomegaly	Supportive management	N/A	Spontaneous resolution after 15 days
Kaman *et al.* [21]	F	4 years	Fever, fatigue, abdominal pain	Icterus, decreased breath sounds on the right side of chest	Supportive management, vit. K	N/A	Spontaneous resolution after 1 week
Vaidya *et al.* [22]	F	7 years	Vomiting, nausea	Icterus, hepatomegaly	Supportive management	N/A	Spontaneous resolution after 2 weeks
Bukulmez *et al.* [23]	F	7 years	Fever, jaundice, abdominal pain	Icterus, hepatomegaly, dullness on the base of right lung, abdominal distention	Supportive management	N/A	Spontaneous resolution after 2 week
Dhakal *et al.* [5]	F	2.5 years	Abdominal pain, scleral icterus	Icterus, hepatomegaly, dullness on the base of right lung	Supportive management	N/A	Spontaneous resolution after 2 week
Hadgu *et al.* [24]	M	4.8 years	Fever, abdominal pain, nausea and vomiting, cough	Bilateral dullness and decreased air entry, hepatomegaly, anicteric	Supportive treatment	N/A	Spontaneous resolution after 1 month
Iza *et al.* [25]	F	32 years	Jaundice epigastric pain, nausea, vomiting, dark urine	Icteric, abdominal tenderness, abdominal distension positive Murphy sign, decreased air entry on right chest	Supportive treatment	N/A	Spontaneous resolution after 4 months

F: Female, M: Male, Y: Years, M: months, N/A: not available, USG: ultrasonography, CXR: chest x-ray, CT: Computed tomography, MRI: Magnetic resonance imaging

**Table 4:** Literature review

Study	Imaging finding	HAV serology	INR	Albumin (g/dl)	Total/direct bilirubin (mg/dl)	Pleural fluid analysis
Saha [15]	USG: bilateral pleural effusion, ascites CXR: left-sided pleural effusion	Serum anti-HAV IgM positive	N/A	2.9	5.6/5	NA
Roy [16] Case 1	USG: hepatomegaly, ascites, bilateral pleural effusion CXR: bilateral pleural effusion (right > left)	Serum and pleural fluid anti-HAV IgM positive	N/A	3.4	2.6/1.4	Total cell count 1500, glucose 99 mg/dl and protein 4.1 g/dl, negative culture
Roy [16] Case 2	USG: hepatomegaly, ascites, bilateral pleural effusion CXR: bilateral pleural effusion (right > left)	Serum anti-HAV IgM positive	N/A	3.2	6.2/6.2	NA
Owen [17]	CXR: right pleural effusion	NA	N/A	N/A	N/A	NA
Dalai *et al.* [14]	USG: right-sided pleural effusion, ascites, hepatomegaly	Serum anti-HAV IgM positive	2	N/A	3.5/1.5	NA
Nagarajan *et al.* [17] Case 1	USG: hepatomegaly, ascites, bilateral pleural effusion CXR: bilateral pleural effusion (right > left)	Serum and pleural fluid anti-HAV IgM positive	N/A	2.5	5.4/4.8	Total cell count 0, protein 20 g/dl
Nagarajan *et al.* [17] case 2	USG: hepatomegaly, bilateral pleural effusion, ascites CXR: bilateral pleural effusion	Serum anti-HAV IgM positive	N/A	3.2	6.9/5.9	NA
Allen *et al.* [18]	USG: ascites, diffuse gallbladder wall thickening CT: ascites, right-side pleural effusion, gallbladder wall thickening	Serum anti-HAV IgM positive	N/A	3.6	6/2.4	NA
Selimoğlu *et al.* [19]	USG: hepatomegaly CXR: right lower lung consolidation	Serum and pleural fluid anti-HAV IgM positive	1.25	3.5	6/3.5	Total cell count 0, glucose 70 mg/dl and protein 4.5 g/dl, negative culture
Mehta *et al.* [13]	CXR: right-side pleural effusion	Serum and pleural fluid anti-HAV IgM positive	N/A	2.8	5.3/5.2	Total cell count 18200, glucose 94 mg/dl and protein 7.7 g/dl, negative culture
Alhan *et al.* [14]	USG: hepatomegaly, right-side pleural effusion	Serum and pleural fluid anti-HAV IgM positive	N/A	4.0	3.9/2.6	Total cell count 0, transudate
Erdem *et al.* [20]	USG: ascites, right-side pleural effusion, thickened gallbladder wall; CXR: right-sided pleural effusion	Serum and pleural fluid anti-HAV IgM, positive	1.1	1.9	6.3/5.6	Total cell count 0, transudate
Ghosh and Kundu [12]	CXR: middle and lower zones of left lung opacity MRI: pleural effusion, hepatosplenomegaly, thickened gallbladder wall	Serum and pleural fluid anti-HAV IgM positive	1.9	N/A	5.6/5.5	Exudative pleural effusion
Gürkan *et al.* [10]	USG: ascites CXR: bilateral pleural effusion	Serum anti-HAV IgM positive	N/A	3.6	6/2.5	NA
Kaman *et al.* [10]	USG: ascites, pleural effusion CXR: right-side pleural effusion	Serum anti-HAV IgM positive	N/A	2.5	6.6/4.8	Glucose 90 mg/dl, negative culture
Vaidya *et al.* [21]	USG: ascites, hepatomegaly, bilateral pleural effusion CXR: left-side pleural effusion	Serum anti-HAV IgM positive	1.1	4	5.2/4.2	NA
Bukulmez *et al.* [22]	USG: hepatomegaly, right-side pleural effusion CT: right pleural effusion	Serum anti-HAV IgM positive	1	3.3	8.2/6.7	NA
Dhakal *et al.* [5]	USG: ascites, bilateral pleural effusion CXR: right-side pleural effusion	Serum anti-HAV IgM positive	N/A	N/A	5.8/4.5	NA
Hadgu *et al.* [24]	USG: mild ascites, hepatosplenomegaly, and small bilateral pleural effusion	Serum anti-HAV IgM positive	1.5	3.8	1.5/0.5	No cells, lactic acid dehydrogenase 15 IU/l, negative TB, negative bacterial culture
Iza *et al.* [25]	USG: right pleural effusion, ascites and acalculous cholecystitis	Serum anti-HAV IgM positive	Normal	3.5	2.6/2.5	N/A

F: Female, M: Male, Y: Years, M: months, N/A: not available, USG: ultrasonography, CXR: chest x-ray, CT: Computed tomography, MRI: Magnetic resonance imaging

All patients were managed supportively. Furthermore, no invasive additional treatments were used in five cases, including intravenous fluids, vitamin K, oral lactulose, and antibiotics prophylactically for bacterial superinfection [12, 14], while thoracostomy and chest tube insertion were only needed in one patient of chylothorax [13].

Of these patients, 95% (19 out of 20 patients) had complete recovery and resolution of pleural effusion and ascites, while one patient (5% of patients) suffered from fulminant liver failure and refractory intracranial pressure increase leading to death 2 weeks after diagnosis [14]

HAV infection is usually self-limited and does not progress to a chronic or latent state, being managed supportively, and the same applies to associated pleural effusions. Pleural effusions do not change the prognosis or require any invasive treatment.

## Conclusion

Pleural effusion is a benign, rare, extrahepatic complication of HAV acute hepatitis, mostly present in juveniles as early right-sided effusion. It resolves spontaneously with supportive management. Thus, further invasive procedures would only complicate this self-resolving benign condition and should be minimized.

## Data Availability

The data used to support the findings of this study are available from the corresponding author upon reasonable request.
